# Editorial: Surface Enhanced Raman Scattering: New Theoretical Approaches, Materials and Strategies

**DOI:** 10.3389/fchem.2020.00063

**Published:** 2020-02-04

**Authors:** Ivano Alessandri, John R. Lombardi

**Affiliations:** ^1^Unit of Research of Brescia, Department of Information Engineering, INSTM, Brescia, Italy; ^2^Department of Information Engineering, Brescia, Italy; ^3^Unit of Brescia, CNR-INO, Brescia, Italy; ^4^Chemistry and Biochemistry Department, City College New Yok, New York, NY, United States

**Keywords:** SERS (surface enhanced Raman scattering), Raman sensing, SERS theory, non-plasmonic SERS forensics, food analysis, biodiagnostics, cultural heritage

The development of surface-enhanced Raman scattering (SERS) has gone hand-in-hand with that of nanotechnology, oscillating between great expectations associated with high sensitivity, which can be extended down to single molecule detection, and frustrating disappointments, originating from low reproducibility of data. This latter critical issue is inherently entangled with the local enhancement of the electromagnetic field, which forms the basis of plasmon-assisted SERS and still prevents its full implementation in analytical chemistry.

In recent years, significant progress has been reported that promises to bring SERS to the forefront of sensing and advanced diagnostics. Several research groups demonstrated that dielectrics can be suitably engineered to achieve non-plasmonic enhanced Raman scattering, reducing invasiveness and increasing reproducibility of SERS experiments (Alessandri, 2013; Alessandri and Lombardi, 2016; Bontempi et al., 2018).

New theoretical models, encompassing plasmonic and non-plasmonic systems, have been developed (Lombardi and Birke, 2014; Roelli et al., 2016; Frizyuk et al., 2018). In parallel, the urgent need for operational SERS-based analysis in complex environments and real-life conditions stimulated the application of multivariate methods to Raman data analysis, opening the door to machine- and deep-learning approaches that are entering the areas of sensing and analytical chemistry on a large scale (Chen et al., 2019; Fan et al., 2019).

This Special Issue includes 16 contributions (1 review, 1 minireview, and 14 research papers) that touch several aspects of SERS, ranging from theory and modeling to analytical applications of both plasmonic and non-plasmonic systems.

In a full review paper, Zou et al. summarized some of the most recent advances in the use of naked and oxide-coated silver nanorods for SERS purposes, giving an overview of their synthesis and applications in different sectors of sensing (food, environment, biological molecules, and gas).

A special focus on the latest developments of shell isolated nanoparticle-enhanced Raman spectroscopy (SHINERS) was reported in the minireview by Krajczewski and Kudelski.

Zhang et al. reported on the simultaneous enhancement of fluorescence emission and Raman scattering achieved by gold-silver nanoalloys coated by silica shells, which opens exciting perspectives for the development of portable devices. Silver nanostars were utilized by de Almeida et al. to classify different types of Portuguese wines on the basis of SERS data processed by principal components analysis. Further specific examples of analytical SERS were treated in research papers dedicated to advanced diagnostics in art, forensics, drug analysis, and biosensing.

Cesaratto et al. described an innovative sample pretreatment based on the use of nitric acid for detecting aniline and xanthene dyes in microscopic specimens of various masterpieces, including a nineteenth-century silk fabric, paper cut-outs by Matisse, Van Gogh's Irises, and Japanese woodblock prints.

Kolhatkar et al. proposed a new method for revealing latent fingermarks based on the combination of SERS and plasmonic luminescence, which exploits the preferential deposition of gold nanoparticles on the biological components of the fingermarks. Another important field that intersects forensics and analytical chemistry is represented by detection of illicit drugs and pharmaceutical compounds. Farquharson et al. provided a detailed SERS library for 39 most common drugs, including opioids, cannabinoids, amphetamines, fentanyl and derived compounds, benzodiazepines, and other psychoactive small molecules. Similar molecules were compared and analyzed on the basis of their chemical structure, pointing out unique Raman bands that can be utilized for identifying and distinguish similar compounds and the modifications to the Raman spectra that can be caused by pH, medium, and the use of different type of metals as enhancers. Muhamadali et al. demonstrated the detection of novel psychoactive substances (methcathinones, aminoindanes, diphenidines, and synthetic cannabinoids) in complex mixtures. Principal component analysis of the SERS data enabled the extension of the detection limit, as well as direct analysis from biological fluids.

In the field of biodiagnostics, Capocefalo et al. synthesized and tested SERS-based nanosensors that are able to discriminate between healthy and cancer cells by measuring their extracellular pH.

Saveleva et al. demonstrated that polycaprolactone fibers coated by porous CaCO_3_ and Ag nanoparticles can selectively adsorb different biological molecules, playing as SERS-active platform for detection of low and high molecular weight molecules.

Finally, Yue et al. utilized nuclear-targeted gold nanorod Raman nanoprobes to investigate *in situ* the dynamic intranuclear molecular changes of a murine melanoma cell line treated with a photosensitizer and irradiated with visible laser (650 nm) in a typical photodynamic therapy experiment, revealing protein degradation and DNA fragmentation.

In the area of theory of SERS, Mueller and Reich presented a model for the plasmonic enhancement mechanism of SERS based on perturbation theory. This model treats SERS as higher-order Raman scattering, allowing the optical absorption cross-section to be calculated and compared with experimental data. This approach clarifies the role of different scattering channels in the case of enhancement factors below 10^3^.

In another research paper, Aranda et al. re-examined the mother of all SERS experiments, i.e., pyridine adsorbed on silver electrodes, to discuss on the use of DFT for modeling the effect of the electrode potential in SERS experiments.

Kim et al. investigated the mechanism of chemical enhancement in non-plasmonic SERS, by testing the Raman response of molecular probes with significant differences in HOMO-LUMO energy gap (4-MPY, 4-MBA, 4-ATP) deposited either on nanostructured (ZnO, GaN) and 2D (WS_2_) semiconductors and excited at different laser wavelengths (488, 514.5, 532, and 632.8 nm). This study shows that the charge transfer from the valence band of the semiconductor to the LUMO of the analyte is the main contribution to the chemical enhancement and can be exploited to maximize the sensitivity of non-plasmonic SERS experiments. This type of charge transfer can be directly controlled by doping, as remarked by Li et al. with an experiment that monitored the variation of Raman intensity of 4-MBA as a function of the Ga content in ZnO nanoparticles utilized as SERS substrates.

In another paper, Ji et al. reported non-plasmonic SERS with ZrO_2_ by direct exploitation of charge transfer, which demonstrates the importance of controlling surface states and extended the results reported by Bontempi et al. on zirconia resonators (Bontempi et al., 2017).

The Editors suggest that such a rich palette of examples, encompassing a large variety of themes, explores the essential features of the multi-faceted scenario of research on SERS, and will serve to guide and inspire future research in the field.

## Author Contributions

IA and JL wrote the manuscript.

### Conflict of Interest

The authors declare that the research was conducted in the absence of any commercial or financial relationships that could be construed as a potential conflict of interest.
